# Exploring the experience of reablement: A systematic review and qualitative evidence synthesis of older people's and carers' views

**DOI:** 10.1111/hsc.13837

**Published:** 2022-05-17

**Authors:** Lachlan Mulquiny, Jodi Oakman

**Affiliations:** ^1^ Centre for Ergonomics and Human Factors, School of Psychology and Public Health La Trobe University Melbourne Victoria Australia

**Keywords:** aged care, carers, home care, qualitative, reablement, review, social care

## Abstract

Concerns from the worldwide ageing population and evidence of poor‐quality aged care services have highlighted the need to develop innovative models of aged care which are acceptable to older people, economically sustainable and are safe. Reablement is a relatively new model for aged care that aims to support older people's desires to age independently in their usual place of residence and decrease dependency on aged care services. This qualitative evidence synthesis aimed to explore the experiences of older people and their carers (formal and informal) towards a reablement model of community aged care to ensure services are considerate of older people's needs. A systematic search was conducted across six electronic databases (Medline, Scopus, CINAHL, PsycINFO, Cochrane Library and Google Scholar) from 1990 to September 2021. Qualitative research exploring older people and their carers' experiences and perceptions of the reablement model used in community aged care services were identified. Nineteen articles were included in the synthesis following the screening of 668 abstracts and 56 full texts. Included articles were subject to quality assessment, and the data were synthesised using thematic synthesis. Three analytical themes were generated from the thematic synthesis; (i) reablement is a shift in approach to aged care, (ii) difficulties in developing tangible and meaningful reablement goals, (iii) reablement improves health and well‐being. Reablement is generally well‐received by older people and their informal carers. However, poor engagement from older people did occur when they had a poor understanding of their role in reablement and when they had not been fully consulted regarding their reablement goals. Current and future reablement services for older people should focus on ensuring an awareness of the processes and principles of reablement and collaboration between practitioner, the older person and their carer when developing goals to increase engagement.

**What is known about the topic**

- Reablement is associated with healthy ageing and aimed at decreasing older people's dependence on long‐term aged care.
- Despite the growing popularity of reablement in aged care services, the clinical effectiveness has not been fully evaluated.

**What this paper adds**

- Older people value the principles of reablement.
- Older people's poor engagement in reablement services can be attributed to a poor understanding of their role in reablement and a lack of collaboration in reablement goals.
- Further research aimed at increasing the motivation and engagement of older people during reablement is required.

## 1 INTRODUCTION

Population ageing is a significant issue and challenges the sustainability of health and social aged care services (Bloom et al., 2015; Harper, 2014). By the year 2050, it is predicted that population ageing will double the cost of long‐term aged care across Organisation for Economic Co‐operation and Development (OECD) countries (Harper, 2014; Martins & de la Maisonneuve, 2006). Traditional aged care services have focussed on long‐term service provision and residential aged care; however, over the past two decades policy makers have shifted their attention towards the implementation of more sustainable models for aged care aimed at maintaining or improving functional skills that lead to decreased reliance on supportive services and demand for residential aged care (Clotworthy et al., 2021; Doh et al., 2020; Metzelthin et al., 2020; Smeets et al., 2020). In Australia, further support for reform in aged care services has arisen due to the recent Australian Commonwealth Government Royal Commission into Aged Care which reported ageism as a systemic issue with negative impacts on the quality of aged care services (Australian Government Royal Commission into Aged Care Quality and Safety, 2021). Moreover, the Commission highlighted multiple failings of the current aged care services, resulting in the delivery of substandard care and harm to the vulnerable older population (Australian Government Royal Commission into Aged Care Quality and Safety, 2021). Suggesting alignment with government aims for aged care services, the reablement model is being widely and increasingly adopted in aged care services across developed nations, including in the United Kingdom, Scandinavia, the United States and New Zealand (Aspinal et al., 2016; Australian Government Department of Health, 2018; Clotworthy et al., 2021; Smeets et al., 2020).

Most older people prefer to maintain a level of independence and social connectivity in their choice of residence and community, which aligns with government policy aimed at reducing the number of older people residing in aged care facilities (Australian Government Productivity Commission, 2011; Boldy et al., 2011; Rostgaard et al., 2011; Wiles et al., 2012). The reablement model of care has been mandated for community aged care delivery across Australia in response to the needs of older people and economic pressures from the ageing population (Australian Government Department of Health, 2018; Australian Government Productivity Commission, 2011). An internationally accepted definition of reablement describes the model as being a person‐centred and goal‐orientated intervention aimed at increasing function, increasing or maintaining independence in a person's place of residence and reducing the need for ongoing services (Metzelthin et al., 2020). Representing a shift from traditional aged care services which have focused on delivering long‐term dependency‐based services typical reablement services offer time limited (generally described as up to 12 weeks) interventions including exercise, behaviour modification and activities of daily living retraining (Lewis et al., 2021; Metzelthin et al., 2017, 2020). Four systematic reviews have investigated the clinical effectiveness of reablement and whether it increases older people's independence and decreases ongoing needs for aged care services. The four reviews identified a small number of primarily poor‐quality articles of mixed or contradictory outcomes; as such the effectiveness of reablement is yet to be fully evaluated (Cochrane et al., 2016; Legg et al., 2016; Sims‐Gould et al., 2017; Tessier et al., 2016).

The experiences of older people who have been involved with reablement are an important consideration so that services can be appropriately tailored to meet their needs. The aim of this systematic review of qualitative literature particularly focuses on the experiences of older people and their carers (formal and informal). The review will explore the experiences, opinions and attitudes towards the reablement model of community aged care. Expanding our understanding is especially relevant at this time when countries are increasingly adopting reablement into their aged care services.

## 2 METHODS

A range of qualitative evidence synthesis (QES) methods are available, and selection is based on multiple factors, including the research question, epistemological assumptions, audience and purpose (Noyes et al., 2018). Based on the objective of our study to understand and evaluate multiple contextual insights from users' experience of the reablement model, we utilised the thematic synthesis method developed by Thomas and Harden (2008). The thematic synthesis approach to QES allows for inductive coding and flexible exploration of data from multiple contexts to interpret and construct meaning from experiences of the phenomena (Barnett‐Page & Thomas, 2009). Our process for identification and selection of articles followed the Preferred Reporting Items for Systematic Reviews and Meta‐Analyses (PRISMA) guidelines (Liberati et al., 2009).

### 2.1 Inclusion and exclusion criteria

We established the inclusion and exclusion criteria prior to screening (see Appendix S1).

#### 2.1.1 Inclusion criteria

We included qualitative studies that were either stand‐alone or included as part of a larger quantitative study or an evaluation study with an identifiable qualitative component. Mixed‐methods studies were included if they separately reported qualitative data collection and analysis. Studies reporting views from a range of experiences and perspectives were included if data between older people and their carers were distinctly identifiable from other, for example, professionals, data. All qualitative design, methodology and analysis techniques were included. Grey literature was also included.

#### 2.1.2 Phenomena of interest

Experiences and perceptions of the reablement model in aged care.

#### 2.1.3 Contexts

The thematic synthesis included studies undertaken where the reablement model has been used to deliver care for older people living in their private residence in their community. We chose this context to maintain some homogeneity of the setting and experience.

#### 2.1.4 Participants

Included studies explored the experiences of the delivery of the reablement model of care from the perspective of the older person receiving care and their carers (formal or informal). For this review ‘older people’ were defined as aged 65 years and older.

#### 2.1.5 Exclusion criteria

Reablement is an approach to aged care that has been introduced over the past three decades, so we limited our search to studies published from 1990 (Clotworthy et al., 2021). Papers were excluded if they were (i) not published in English, (ii) literature reviews, (iii) based solely on quantitative data and (iv) based solely on the perspectives of healthcare professionals. Studies with participants who permanently resided in long‐term aged care facilities and who were provided care 24 h a day, 7 days a week, were also excluded in line with the aim of the study which was centred on older adults living in the private home setting in their local community.

### 2.2 Search strategies

An exploratory search of the Medline database was undertaken to identify relevant articles for inclusion in the review. Studies identified in this exploratory phase were used to expand search terms, MESH terms and identify appropriate keywords. Using these search terms, we searched the following databases: Medline, Scopus, CINAHL, PsycINFO, Cochrane Library and Google Scholar. The timeframe of our initial search covered from 1990 to December 2019. At a later stage, we updated this search focusing on papers published between 2019 and September 2021. Database searches were limited to the English language.

### 2.3 Study selection process

**FIGURE 1:**
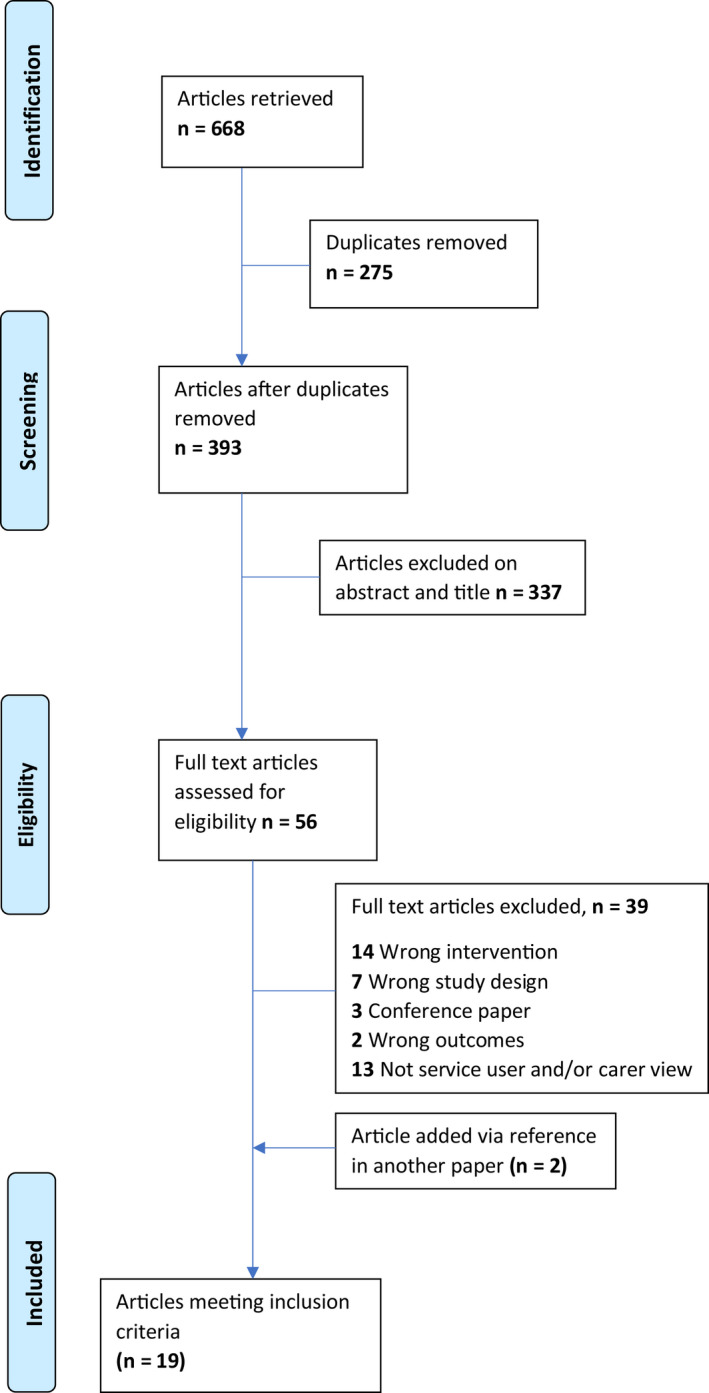
Modified PRISMA flow diagram of the systematic search strategy (Moher et al., 2009). PRISMA, Preferred Reporting Items for Systematic Reviews and Meta‐Analyses

Studies were exported from each database into EndNoteX9 (EndNote, 2018) and then transferred to the Covidence software package (Covidence, 2019), which was used to manage the screening and selection process. Abstracts were screened by both reviewers using a priori inclusion and exclusion criteria (see Appendix S1). Conflicts were resolved through discussion between both reviewers. Following abstract screening, full texts were reviewed in Covidence, with conflicts resolved through discussion. The reference lists of included studies were also screened. Full screening details are presented in the PRISMA diagram (Figure 1).

### 2.4 Appraisal of study quality

All studies were appraised using the Joanna Briggs Institute Critical Appraisal Checklist for Qualitative Research (JBI QARI) (Lockwood et al., 2015). The JBI QARI is a 10‐item instrument used for the quality assessment of qualitative research. Items are assessed as ‘yes’, ‘no’, ‘unclear’ or ‘not applicable. The checklist assesses for congruence between philosophical perspectives, methodology selection, data analysis methods, interpretation of results and conclusions and that participant voices are adequately represented.

### 2.5 Data extraction, analysis and synthesis

Data extraction and synthesis of the included papers followed the ‘thematic synthesis’ method, a three‐step process developed and described by Thomas and Harden (2008). All included papers were imported into NVIVO 12 Pro (NVivo, 2018). Results sections were then inductively coded line‐by‐line to produce ‘free codes’ which summarise and capture the meaning of the published text (Thomas & Harden, 2008). Free codes with similar meaning were then organised together into groups which were then labelled with new codes that described the shared meaning of the free codes. This process helped with translating similar concepts found across the included papers into more manageable data used to develop analytical themes. Until this point, the developed themes had been firmly rooted in the primary studies and not considered in relation to the review objective. Therefore, in the third stage, the descriptive themes were analysed, and their relevance considered against the review questions to generate analytical themes. Throughout all three stages of the process, reference to primary data source was undertaken to ensure that generated ideas were accurately representing the data. To test for internal validity of the generated analytical themes, coding from a random sample of included studies using the final framework was completed by Jodi Oakman (an experienced qualitative researcher).

### 2.6 Reflexive note

Analysis of the data was conducted within the epistemological stance of critical realism, which assumes that our understanding of reality is mediated by our beliefs and perceptions (Barnett‐Page & Thomas, 2009). Critical realism includes elements of both realist and constructionist paradigms, acknowledging individuals' different perspectives in their understanding of reality (Braun & Clarke, 2013; Chouliaraki, 2002; Kuhn & Westwell, 2012). We used this approach as critical realists seek to make recommendations for policy and practice, which are based on an explanation of individuals' tendencies and causal mechanisms (Fletcher, 2017). The main analysis was conducted by one researcher Lachlan Mulquiny, who has a background in podiatry. The initial themes and the analysis were discussed and reviewed by the co‐author Jodi Oakman who is an experienced practitioner and researcher with a background in physiotherapy, ergonomics and human factors.

## 3 RESULTS

### 3.1 Search strategy and study selection

Our initial database search identified 10 articles that met the inclusion criteria. From the September 2021 database search, a further seven articles were identified as meeting the inclusion criteria. Two articles were added after being identified through reference checking the included articles. A total of 19 articles were included for appraisal and synthesis. The PRISMA diagram is shown in Figure 1.

Five countries were represented across the 19 included articles: England (Beresford et al., 2019; Chung, 2019; Glendinning et al., 2010; Trappes‐Lomax & Hawton, 2012; Whitehead et al., 2018; Wilde & Glendinning, 2012), Norway (Bødker et al., 2019; Hjelle, Alvsvag, et al., 2017; Hjelle, Tuntland, et al., 2017; Jakobsen et al., 2019; Jokstad et al., 2020; Magne & Vik, 2020; Moe & Brinchmann, 2016), Scotland (Ghatorae, 2013; McLeod & Mair, 2009), Australia (Golenko et al., 2021; Jeon et al., 2019; Rahja et al., 2020) and Sweden (Östlund et al., 2019). The median date of publication across the studies was 2018 (range 2009–2021), suggesting this is a relatively new field of research. There were five articles (Beresford et al., 2019; Ghatorae, 2013; Glendinning et al., 2010; Golenko et al., 2021; McLeod & Mair, 2009) that included qualitative data from the perspective of reablement service professionals; however, the data from these individuals was clearly distinguishable and was excluded from the thematic synthesis. Two papers reported on data collected during the same study (Glendinning et al., 2010; Wilde & Glendinning, 2012). Both papers have been included in the thematic synthesis given their varied reporting styles, including quotations and exploration of concepts, which we believe improved the richness of the dataset used for synthesis. Further characteristics of the included studies are provided in Table 1.

**TABLE 1:** Overview of studies included in the QES

First author, year (Country)	Participants	Data collection method	Data analysis approach	Focus of study
Beresford, 2019 (England)	Service users *n* = 31 Carers of people receiving a reablement service *n* = 2 Service professionals = 32	Semi‐structured interview	Thematic analysis	An evaluation of reablement services in England used to develop a service model typology. The evaluation utilised mixed methods to investigate outcomes, factors which impact on outcomes and user and practitioner's views
Bødker, 2019 (Denmark)	Service users *n* = 8	Ethnographic fieldwork including observation (n = 8) and semi‐structured interviews (n = 4)	Abductive analysis characterised by interactive and iterative approach to analysis	Merging a conceptual critique of reablement with an empirical exploration of older people's experiences in reablement services
Chung, 2019 (England)	Service users *n* = 16	Semi‐structured interview	Grounded theory	Explore the experience of enablement services from people living with dementia
Ghatorae, 2013 (Scotland)	Service users *n* = 13 Carers of people receiving a reablement service *n* = 10 Service professionals = 56	Multiple semi‐structured interviews collected during the reablement period	Not reported	A mixed‐methods evaluation of the Glasgow City Council reablement programme with the qualitative research examining the impact of reablement on stakeholders in terms of satisfaction, the local processes, and arising issues
Glendinning, 2010 (England)	Service users *n* = 34 Carers of people receiving a reablement service *n* = 10 Service professionals = 40	Semi‐structured interview	Thematic analysis through a priori framework and emergent themes from interviews	For service users: factors which influenced reablement progress and outcomes for service users themselves and informal carers (if any) Carers: explore informal carers' experiences of helping service users and the impact of home care reablement service on the care‐giving role
Golenko, 2021 (Australia)	Service users *n* = 18 Service professionals = 8	Semi‐structured interview	Thematic analysis	What was the experience of older people in a wellness and reablement programme and how did it impact on their health and well‐being?
Hjelle, Alvsvag, 2017 (Norway)	Carers of people receiving a reablement service *n* = 6	Semi‐structured interview	Systematic text condensation	Carers experiences with participation in reablement processes
Hjelle, Tuntland, 2017 (Norway)	Service users *n* = 8	Semi‐structured interview	Content analysis	Describe how older adults experience participation in reablement, with focus on interaction and finding motivation
Jakobsen, 2019 (Norway)	Carers of people receiving a reablement service *n* = 15	Semi‐structured interview	Constructivist grounded theory	How adult children perceive the collaboration of older parents, other family members and healthcare professionals in reablement services
Jeon, 2019 (Australia)	Service users *n* = 7	Semi‐structured interview	Content analysis	To explore the overall experience of I‐HARP and key barriers and facilitators to its implementation. To determine the potential impact of I‐HARP on clients' daily activities, mobility and health‐related quality of life, mood and caregiver burden and quality of life
Jokstad, 2020 (Norway)	Service users *n* = 10	Multiple semi‐structured interviews collected at the start of reablement, end of reablement, and 6 weeks post reablement	Thematic content analysis	Explore and described older peoples involvement in reablement from the beginning of the period until 6 weeks post the intervention
Magne, 2020 (Norway)	Service users *n* = 10	Semi‐structured interview	Systematic text condensation	Explore how older people experience reablement and opportunities to participate in daily activities
McLeod, 2009 (Scotland)	Service users *n* = 14 Service professionals = 16	Semi‐structured interview	Not reported	Ascertain the views of service users of a new reablement service
Moe, 2016 (Norway)	Service users *n* = 17 Carers of people receiving a reablement service *n* = 10	Semi‐structured interview	Grounded theory	The service users experience of reablement services to generate a grounded theory
Östlund, 2019 (Sweden)	Service users *n* = 23	Semi‐structured interview	Content analysis	Explore older people's experiences of the reablement model in terms of autonomy in life and the importance of significant others during the reablement period
Rahja, 2020 (Australia)	Service users *n* = 5 Carers of people receiving a reablement service *n* = 10	Semi‐structured interview	Thematic analysis	To explore and describe the experiences and outcomes from participating in a dementia reablement program
Trappes‐Lomax, 2012 (England)	Service users *n* = 42	Semi‐structured interview	Interpretative phenomenological analysis	Describe the experience of participants involved with a reablement informed rehabilitation service with reference to what worked well and what did not work well
Whitehead, 2018 (England)	Service users *n* = 5	Semi‐structured interview	Thematic analysis	Acceptability of a reablement service delivered by Occupational Therapists
Wilde, 2012 (England)	Service users *n* = 34 Carers of people receiving a reablement service *n* = 10	Semi‐structured interview	Thematic analysis through a priori framework and emergent themes from interviews	Exploration of service users' experiences of reablement, the outcomes for the users themselves, their perceptions of the ways reablement had affected any informal carers; and any outstanding unmet needs

Abbreviation: QES, qualitative evidence synthesis.

### 3.2 Quality appraisal

Table 2 presents the quality appraisal of each study using the JBI QARI (see Appendix S2 for JBI QARI questions). Of the included studies, 17 were published in peer‐reviewed journals. The remaining two studies were identified from the grey literature, and both had an adequate description of the study methods to complete an appraisal using the JBI QARI.

**TABLE 2:** Appraisal of study quality using JBI QARI

First author (year)	Q1	Q2	Q3	Q4	Q5	Q6	Q7	Q8	Q9
Beresford (2019)	No	Yes	Yes	Yes	Yes	No	Yes	Yes	Yes
Bødker (2019)	Yes	Yes	Yes	Yes	Yes	No	No	Yes	Yes
Chung (2019)	Yes	Yes	Yes	Yes	Yes	No	Yes	Yes	Yes
Ghatorae (2013)	No	Yes	Yes	Yes	Yes	No	No	Yes	Unclear
Glendinning (2010)	Unclear	Yes	Yes	Yes	Yes	No	No	Yes	Yes
Golenko (2021)	Yes	Yes	Yes	Yes	Yes	No	No	Yes	Yes
Hjelle, Alvsvag (2017)	Yes	Yes	Yes	Yes	Yes	No	Yes	Yes	Yes
Hjelle, Tuntland (2017)	Yes	Yes	Yes	Yes	Yes	No	Yes	Yes	Yes
Jakobsen (2019)	No	Yes	Yes	Yes	Yes	No	Yes	Yes	Yes
Jeon (2019)	No	Yes	Yes	Yes	Yes	No	No	Yes	Yes
Jokstad (2020)	No	Yes	Yes	Yes	Yes	No	No	Yes	Yes
Magne (2020)	No	Yes	Yes	Yes	Yes	No	No	Yes	Yes
McLeod (2009)	No	Yes	Yes	Unclear	Yes	No	Yes	Yes	Unclear
Moe (2016)	Yes	Yes	Yes	Yes	Yes	No	Yes	Yes	Yes
Östlund (2019)	Unclear	Yes	Yes	Yes	Yes	No	No	Yes	Yes
Rahja (2020)	No	Yes	Yes	Yes	Yes	No	Yes	Yes	Yes
Trappes‐Lomax (2012)	Yes	Yes	Yes	Yes	Yes	No	Yes	Yes	Yes
Whitehead (2018)	Unclear	Yes	Yes	Yes	Yes	No	Yes	Yes	Yes
Wilde (2012)	Unclear	Yes	Yes	Yes	Yes	No	No	Yes	Yes

Abbreviation: JBI QARI, Joanna Briggs Institute Critical Appraisal Checklist for Qualitative Research.

### 3.3 Thematic synthesis

Three analytical themes were generated from the synthesis of findings from the included studies: reablement is a shift in approach to aged care; difficulties in developing tangible and meaningful reablement goals; reablement improves health and well‐being.

#### 3.3.1 Theme 1—Reablement is a shift in approach to aged care

The aims and processes of reablement, with a focus on restoring lost capacities to improve older people's ability to perform activities for daily living, were often misunderstood by older people and their carers who were expecting services that would compensate for their losses in function.

##### Misunderstanding of reablement

Reablement presents a model which promotes active engagement from the older person to regain functional losses to help maintain their independence. However, across the included studies, older people participating in reablement programmes reported that the services received had not been in line with their expectations (Beresford et al., 2019; Bødker et al., 2019; Hjelle, Alvsvag, et al., 2017; Jokstad et al., 2020; McLeod & Mair, 2009; Rahja et al., 2020; Wilde & Glendinning, 2012). Older people's expectations of reablement typically reflected ideas from more traditional aged care services which focused on others engaged to undertake tasks for the older person; which contrasts with a more hands‐off and rehabilitative practice (Beresford et al., 2019; Bødker et al., 2019; Chung, 2019; Ghatorae, 2013; Glendinning et al., 2010; Jokstad et al., 2020; Magne & Vik, 2020; Moe & Brinchmann, 2016; Rahja et al., 2020; Wilde & Glendinning, 2012). The following quote illustrates an older person's expectation of more traditional aged care services:

‘I didn't’ understand all of it. I don't suppose anybody‐ I was just told I'd get a carer coming in, in the morning, to come and help me get washed and dressed’ (Wilde & Glendinning, 2012).

This misunderstanding of the aims of reablement did result in dissatisfaction with reablement services leading to resistance and had the potential to negatively impact reablement outcomes (Beresford et al., 2019; Glendinning et al., 2010; Hjelle, Tuntland, et al., 2017; Jokstad et al., 2020; Wilde & Glendinning, 2012). For instance, older people were surprised when reablement workers came to their house, prescribed exercises and adjusted occupational routines aimed at maximising function in the performance of routine tasks (Beresford et al., 2019; Bødker et al., 2019; Hjelle, Tuntland, et al., 2017; Rahja et al., 2020; Wilde & Glendinning, 2012). In general, the understanding of reablement improved during participation in reablement services (Beresford et al., 2019; Hjelle, Tuntland, et al., 2017; Jokstad et al., 2020; Magne & Vik, 2020; Moe & Brinchmann, 2016; Trappes‐Lomax & Hawton, 2012; Whitehead et al., 2018). Finally, older people and their carers expected the reablement service to continue indefinitely. Some older people and their carers described the ending of the reablement period as abrupt and suggested a need for a smoother transition at the end of the intervention period. Older people and their carers reported feeling stressed and anxious about their future when services were reduced (Beresford et al., 2019; Bødker et al., 2019; Hjelle, Alvsvag, et al., 2017; Jakobsen et al., 2019; McLeod & Mair, 2009; Östlund et al., 2019; Whitehead et al., 2018).

#### 3.3.2 Theme 2—Developing reablement goals

Developing goals for the older person are a fundamental step in the reablement process. A key finding across the included studies was that older people and their carers identified goals as an important measure and motivator during the reablement process and attainment of goals had a positive impact on their experience (Chung, 2019; Hjelle, Alvsvag, et al., 2017; Jokstad et al., 2020; Magne & Vik, 2020; Wilde & Glendinning, 2012). Conversely, the inability to attain goals may be discouraging for older people, resulting in a negative experience of the reablement period (Golenko et al., 2021; Wilde & Glendinning, 2012). Across the studies many older people believed that their goals were not negotiated at commencement of the reablement period with only a few older people able to recall goals after their reablement period had concluded (Beresford et al., 2019; Jokstad et al., 2020; McLeod & Mair, 2009; Wilde & Glendinning, 2012):

‘They may have had goals I don't know, but I don't remember talking to them about them’ (Beresford et al., 2019).

When older people were able to recall their reablement goals, the focus was primarily on improving functional capacity for the completion of activities of daily living, with no consideration of social engagement (Beresford et al., 2019; Bødker et al., 2019; Chung, 2019; Golenko et al., 2021; McLeod & Mair, 2009; Östlund et al., 2019; Rahja et al., 2020; Wilde & Glendinning, 2012). A difficulty for many older people was linking the reablement interventions, such as exercises, with the goals of their reablement (Beresford et al., 2019; Jokstad et al., 2020; McLeod & Mair, 2009; Wilde & Glendinning, 2012). In some circumstances the priorities of older people were not considered, and goals were imposed that did not align with their lifestyle preferences leading to decreased engagement during the reablement period. (Bødker et al., 2019; Chung, 2019; Golenko et al., 2021; Moe & Brinchmann, 2016; Rahja et al., 2020). Further, goal setting was often seen as a ‘new skill’ and a difficult task which was not appropriate for them given their age:

‘I think achieving my goals is history. I'm 73 years of age’ (Golenko et al., 2021).

##### Caregivers perspectives for goal setting

Carers were often excluded from the reablement process despite their wish to be involved (Glendinning et al., 2008; Hjelle, Alvsvag, et al., 2017; Jakobsen et al., 2019; Wilde & Glendinning, 2012). Carers voiced concerns of being overlooked as a resource to provide alternative perspectives and insights into the current health status of the older person, which could be overlooked (Hjelle, Alvsvag, et al., 2017; Jakobsen et al., 2019). Tensions between older people and their carers (most often family members) arose when views and perspectives diverged in the development of attainable reablement goals (Hjelle, Alvsvag, et al., 2017). Some carers felt that due to their responsibilities in the care‐giving relationship, including navigating various aspects of care and assistance with everyday tasks, they could provide helpful input into the development of strategies to assist with achieving reablement goals (Hjelle, Alvsvag, et al., 2017; Moe & Brinchmann, 2016). The following quote from a family member illustrates these concerns:

‘We discovered that one of the goals of our family member's reablement was to go down the basement stairs to wash clothes. As family, we disagreed with this goal because in our opinion, the basement stairs are steep and dangerous for her. We wanted her to have a washing machine in the kitchen. I think it is necessary and important that the reablement service collaborate with the relatives and request their opinion of the goals’ (Hjelle, Alvsvag, et al., 2017).

##### Individual independence

Older people reported their achievements during the reablement period were appropriate for ‘in the home’ but the programme did not address their social isolation (Beresford et al., 2019; Bødker et al., 2019; Moe & Brinchmann, 2016; Östlund et al., 2019; Wilde & Glendinning, 2012). Many older people voiced concerns that reablement services tended to narrowly define independence as not receiving home support to complete activities for daily living, and this placed limitations on how they could define their goals (Beresford et al., 2019; Bødker et al., 2019; Östlund et al., 2019; Trappes‐Lomax & Hawton, 2012; Wilde & Glendinning, 2012). Despite the importance of leisure activities and having a social life for older people living in the community, these factors were reported as being rarely included in the goals of reablement programmes (Beresford et al., 2019; Bødker et al., 2019; Östlund et al., 2019; Wilde & Glendinning, 2012).

#### 3.3.3 Theme 3—A sense of improved health and well‐being

The following sub‐themes explore the improvements in health and well‐being from the perspectives of older people and their carers.

##### Older persons improved health and well‐being

Many older people had a positive opinion of the underlying principles of reablement, particularly its focus on enabling them to maintain independence in their usual residence and promotion of active participation (Bødker et al., 2019; Chung, 2019; Glendinning et al., 2010; Hjelle, Tuntland, et al., 2017; Magne & Vik, 2020; McLeod & Mair, 2009; Moe & Brinchmann, 2016; Rahja et al., 2020; Whitehead et al., 2018; Wilde & Glendinning, 2012). For many older people, their improved ability to complete personal care and meal preparation increased their self‐confidence, motivating them to seek improvements across other areas of their daily life (Beresford et al., 2019; Chung, 2019; Ghatorae, 2013; Jokstad et al., 2020; Magne & Vik, 2020; Östlund et al., 2019; Rahja et al., 2020). Encouragement to improve and be considered capable by someone from outside their family was recognised as highly motivating by the older person (Beresford et al., 2019; Hjelle, Tuntland, et al., 2017; Magne & Vik, 2020; Moe & Brinchmann, 2016).

‘… it's just the fact that another human being comes to see you that makes you feel better and gives you the bit of confidence’ (Beresford et al., 2019).

However, it was common that at the end of the reablement period, some older people did not continue with activities to maintain the gains made during the programme. One reason reported by older people for abandoning the activities and exercises undertaken during reablement was a lack of support or encouragement once the official service had ended (Bødker et al., 2019; Hjelle, Tuntland, et al., 2017; Jokstad et al., 2020). For some older people, the ending of reablement was associated with anxiety as they were concerned that there would be negative impacts on their health and function without a connection to their reablement clinicians (Bødker et al., 2019; McLeod & Mair, 2009; Whitehead et al., 2018; Wilde & Glendinning, 2012).

##### Improved carer well‐being

Carers reported improved confidence through observing reablement clinicians and developing new and more structured caring skills (Jeon et al., 2019; Wilde & Glendinning, 2012). These newly acquired skills helped them support older people's performance and assist them with sustaining improvements made during the reablement period (Glendinning et al., 2010; Hjelle, Alvsvag, et al., 2017; Wilde & Glendinning, 2012). The impact of improving their family member's independence resulted in benefits for carers who now had additional free time for their own everyday life activities (Glendinning et al., 2010; Hjelle, Alvsvag, et al., 2017; Jeon et al., 2019; Wilde & Glendinning, 2012).

## 4 DISCUSSION

This review is the first QES to explore the experiences of older people and their informal care networks receiving aged care in the community informed by the reablement model. Traditional aged care services have typically been focussed on delivery of domestic services where older people are passive recipients. In contrast, delivery of reablement is aimed at increasing self‐sufficiency and independence in the home through active engagement from the older person (Aspinal et al., 2016; Jacobi et al., 2020). Our synthesis found that confusion and misunderstanding of the aims and process of reablement negatively impacted older persons engagement and participation during their reablement process (Beresford et al., 2019; Bødker et al., 2019; Ghatorae, 2013; Hjelle, Tuntland, et al., 2017; Jokstad et al., 2020; McLeod & Mair, 2009; Rahja et al., 2020; Wilde & Glendinning, 2012). Therefore, as suggested by Stausholm et al. (2021), it is important for clinicians to effectively communicate the aim and content of reablement in an understandable way to maximise engagement and improve collaboration during the goal development phase.

Goals were often viewed as an important motivator to improve independence and were a means of ‘looking forward to be the person they were before’ (Hjelle, Tuntland, et al., 2017). Hjelle, Alvsvag, et al. (2017) reported that tensions arose between older people and those in their informal care networks regarding the appropriateness and prospect of achieving goals identified by the older person. Moreover, some family members felt overlooked in the goal setting stage and reported that they would appreciate an invitation to provide further information and perspective of their family members current situation to help contribute to possible solutions (Hjelle, Alvsvag, et al., 2017). This experience is not unique to the reablement model of care, and Plant et al. (2016) have previously suggested that successful goal setting in these circumstances includes building consensus between the service participant and their family, to ensure that goals are holistic and not unrealistic. In practice, this involves open dialogue where the older person, their carers and clinicians can share knowledge and different perspectives regarding the appropriateness and achievability of goals and arrive at a common understanding (Legare et al., 2011; Siegert & Taylor, 2004).

Both older people and their carers identified the setting of goals was an important indicator for success in reablement, but only when the older person felt that consultation was adequate and goals were congruent with their meaning of independence (Beresford et al., 2019; Chung, 2019; Golenko et al., 2021; Hjelle, Tuntland, et al., 2017; Jokstad et al., 2020; McLeod & Mair, 2009; Wilde & Glendinning, 2012). Our synthesis found the definition of independence was frustrating for older people when it was narrowly defined as completion of activities of daily living and did not include their social needs (Beresford et al., 2019; Bødker et al., 2019; Östlund et al., 2019; Wilde & Glendinning, 2012). Some older people reported that their goals to be more socially active were not considered to guide their reablement, instead, the goals of their reablement tended to reflect the priorities of governments and local aged care services to deliver short‐term interventions aimed at decreasing user dependency on ongoing supportive services (Beresford et al., 2019; Bødker et al., 2019; Östlund et al., 2019; Wilde & Glendinning, 2012). This perception has also been reported by reablement professionals who described a tension between local government priorities to reduce demand on social services and disinterest from older people to do some tasks (e.g. home cleaning, bathing) for themselves (Stausholm et al., 2021).

The issues of social isolation and loneliness in older people are well documented (Gardiner et al., 2018; Luanaigh & Lawlor, 2008). Social isolation and loneliness in older age have been associated with depression, decreased cognitive function, increased cardiovascular disease and mortality (Courtin & Knapp, 2015). The evidence from this synthesis suggests that reablement interventions do little to address older people's needs regarding increased social connectedness. Recent studies have examined the content of reablement interventions, which have focussed on improving body function and overlooked the meaningfulness and transferability in relating the prescribed exercises to participation in social activities (Doh et al., 2020; Eliassen & Lahelle, 2021; Mjosund et al., 2021). Current reablement practices for older people appear to focus on physical health with limited attention on mental health. As suggested by Doh et al. (2020), we also encourage reablement services for older people to place greater attention on increasing social connectivity.

The ending of reablement was described by both older people and their carers as abrupt, and that sustaining the functional gains made during the period was difficult to achieve (Beresford et al., 2019; Hjelle, Alvsvag, et al., 2017; Jokstad et al., 2020; Östlund et al., 2019). Hjelle, Alvsvag, et al. (2017) reported concern from carers of a missing link that would help sustain functional gains and suggested a smoother transition was needed at the end of the reablement programme. In addition to insufficient follow‐up, when older people were referred to services following the reablement programme, it often involved a return to dependent models of care with limited scope to sustain functional gains made during their reablement period (Bødker et al., 2019; Hjelle, Alvsvag, et al., 2017; Whitehead et al., 2018). These findings suggest that older people and their carers considered reablement an ongoing process that cannot be limited to short‐term programmes (Beresford et al., 2019; Bødker et al., 2019; Östlund et al., 2019; Whitehead et al., 2018; Wilde & Glendinning, 2012). Carers believed that care services delivered after reablement were orientated towards older people being passive recipients of dependent care and would hinder sustaining the gains made during reablement, leading to another decline and return to pre‐reablement levels of function.

Positive experiences of receiving reablement interventions included the emergence of a more optimistic outlook and empowerment that supported progress towards the older person's independence, allowing the older person to maintain independence within their usual place of residence (Beresford et al., 2019; Magne & Vik, 2020). Positive experiences are consistent with the intent of the reablement model and with goals set in aged care policy across developed countries and the World Health Organization, particularly the goal to facilitate older people to continue to remain in their usual place of residence and delay admission to long‐term aged care facilities (Aspinal et al., 2016; Rudnicka et al., 2020; World Health Organization, 2017).

### 4.1 Strengths and limitations

We conducted a comprehensive literature search of six electronic databases and screened reference lists of included studies. We took the additional step to include grey literature to maximise eligible studies. Both reviewers were involved in the screening, data extraction and quality appraisal stages of the review. Some limitations of this review were noted, such as limiting the search strategy to English which may have resulted in the omission of some relevant papers. Second, inherent with qualitative research, we acknowledge that thematic analysis of the data may be interpreted differently by reviewers with different backgrounds and experiences.

## 5 CONCLUSION

This review of qualitative studies explored the experiences of older people and their carers who had participated in reablement informed community aged care. Older people and their carers value the underlying principles of reablement to support independence in their usual place of residence. However, some older people found that the short time periods for reablement services were insufficient for their level of disability and considered a need for ongoing interventions. Older people also felt their reablement goals tended to ignore their need for social connectedness.

Improvements to reablement services should focus on developing goals in collaboration with older people and their informal care networks and seek to include the psychosocial needs of older people, as both strategies should increase the engagement of older people. Older people need to be educated on the reablement process and what is required as participants in the programme. Aged care providers could consider redesigning their services to integrate reablement principles.

Future research should focus on evaluating the effectiveness of reablement on reducing demand for ongoing aged care services and identifying the motivators and strategies to improve engagement of older people during their reablement period.

## CONFLICT OF INTEREST

There is no conflict of interest to declare.

## Supporting information


Appendix S1

Appendix S2

## Data Availability

The data that support the findings of this study are available from the corresponding author upon reasonable request.
