# Supplementary material for: Exploring the experience of reablement: A systematic review and qualitative evidence synthesis of older people's and carers' views
Source: Health Soc Care Community. 2022 May 17;30(5):e1471–83. doi: 10.1111/hsc.13837 (PMC9540535; doi:10.1111/hsc.13837)
Supplement: Supplementary file 1 — Appendix S1 [file HSC-30-e1471-s002.docx]

| **Appendix 1: Inclusion/exclusion criteria and their justification** | | | |
| --- | --- | --- | --- |
| **Item** | **Inclusion criteria** | **Exclusion criteria** | **Main justification** |
| Time frame | Studies published from 1990 onwards | Studies published before 1990 | The research on the older persons’ experiences of receiving social care emerged in the nineties |
| Location and Language | Studies from any location were included, as long as they were published in English | Studies not written in English | Studies location was not restricted in order to ensure no relevant studies were missed.  English is the most familiar language for the reviewers. |
| Population | Studies focused on either (or both)  - older people (people aged 65 and over), living in the community  - formal and/or informal carers of older people receiving care  … who were receiving care informed by the re-ablement model (or who had received it within the past 12 months) at the moment of data collection. | Studies focused on people under 65 years old  Studies focused on older people living in an institutional setting  Studies focused on older people receiving community care not informed by the re-ablement model.  Studies focused on carers of older people receiving community care not informed by the re-ablement model.  Older people (and older people’s carers) who had experienced the re-ablement model more than 12 months ago. | Western societies tend to associate 65 with the beginning of old age  Only studies of older people living in the community were included in the literature review because, currently in Australia, re-ablement is only focussed on older people living in the community.  We decided to admit the inclusion of studies whose older persons were not receiving care at the moment of data collection in order to be less restrictive. We limited the past experience of receiving care to a maximum period of one year from the moment of data collection (in order to minimise the risk of memory loss). |
| Outcome | Studies reporting the accounts from older people and/or older peoples carers (both formal and informal) of their experiences and perspectives of receiving re-ablement care. | Studies not reporting the accounts from older people and/or older peoples carers (both formal and informal) of their experiences and perspectives of receiving re-ablement care. | Data collected directly from the population under investigation provides a better account of their experiences and perspectives than data collected from other people |
| Research type | Studies based qualitative research (data collection and analysis)  Qualitative methods could be combined with quantitative methods | Studies not based on qualitative research  Literature reviews, opinion articles | Qualitative research is the most suitable research approach to capture experiences and perspectives in the own words of the research participants |
| Publication type | Articles, books, book chapters, dissertations, research reports, policy documents (which included description of study design) | Website documents, media articles and other non-research documents. | A broad range of research informed publication types were included in order collect as many papers as possible as qualitative research in this area is known to be at current quite scarce.  Website documents, media articles and other non-research documents were excluded because they do not offer research-based evidence. |
