# Supplementary material for: Exploring the experience of reablement: A systematic review and qualitative evidence synthesis of older people's and carers' views
Source: Health Soc Care Community. 2022 May 17;30(5):e1471–83. doi: 10.1111/hsc.13837 (PMC9540535; doi:10.1111/hsc.13837)
Supplement: Supplementary file 2 — Appendix S2 [file HSC-30-e1471-s001.docx]

# Appendix -2 : JBI Critical Appraisal Checklist for Qualitative Research

Questions are answered either Yes, No, Unclear, or Not applicable

1. Is there congruity between the stated philosophical perspective and the research methodology?
2. Is there congruity between the research methodology and the research question or objectives?
3. Is there congruity between the research methodology and the methods used to collect data?
4. Is there congruity between the research methodology and the representation and analysis of data?
5. Is there congruity between the research methodology and the interpretation of results?
6. Is there a statement locating the researcher culturally or theoretically?
7. Is the influence of the researcher on the research, and vice- versa, addressed?
8. Are participants, and their voices, adequately represented?
9. Is the research ethical according to current criteria or, for recent studies, and is there evidence of ethical approval by an appropriate body?
10. Do the conclusions drawn in the research report flow from the analysis, or interpretation, of the data?
